# Prevalence, Causes, and Risk Factors Associated with Visual Impairment in Qbah, a Rural Community in the Qassim Region of Saudi Arabia

**DOI:** 10.3390/healthcare13040426

**Published:** 2025-02-16

**Authors:** Sulaiman Aldakhil, Saif Hassan Alrasheed, Raghda Faisal Mutwaly, Bandar Alenezi, Saad Alrabiah, Mohammed M. Alnawmasi, Nawaf M. Almutairi, Saja A. Alhoshan, Bashair N. Alnasser

**Affiliations:** 1 Department of Optometry, College of Applied Medical Sciences, Qassim University, Buraydah 51452, Saudi Arabia; s.dakhil@qu.edu.sa (S.A.); r.mutwaly@qu.edu.sa (R.F.M.); b.alenezi@qu.edu.sa (B.A.); nm.almutari@qu.edu.sa (N.M.A.); 2 Department of Ophthalmology, King Fahad Medical City, Riyadh 12231, Saudi Arabia; saad_r@windowslive.com; 3 Department of Ophthalmology, King Abdulaziz Medical City, National Guard Health Affairs, Riyadh 11426, Saudi Arabia; 4 Department of Ophthalmology, Armed Forces Hospital, Armed Forces Medical Services, Al-Kharj 16274, Saudi Arabia; bashair.nas@gmail.com

**Keywords:** visual impairment, rural community, risk factor, public health, age factors

## Abstract

Background: Visual impairment can significantly impact an individual’s performance, productivity, and overall quality of life. Objectives: The objective of this paper is to report the prevalence, causes, and risk factors associated with visual impairment in Qbah, a rural community in the Qassim Province of Saudi Arabia. Methods: A descriptive cross-sectional study was conducted in Qbah, a rural community, with 587 subjects aged 6 to 80 years. Data were collected as part of a major campaign by Qassim University, and each participant underwent a comprehensive eye examination. Finally, the visual impairment was classified based on the International Classification of Diseases 11th revision, 2018 (ICD-11). Results: The overall prevalence of presenting visual impairment was 159 (27.1%). This included mild visual impairment 84 (14.37%), moderate visual impairment 65 (11.7%), severe visual impairment, and blindness 5 (0.90%). The main causes of visual impairment were uncorrected refractive errors (UREs), at 132 (83.0%), followed by amblyopia, at 14 (8.8%), and cataract, at 9 (5.7%). The prevalence of hyperopia, myopia, and astigmatism was 134 (22.8%), 174 (29.6%), and 13 (2.2%), respectively. Regression analysis revealed that the odds for visual impairment were 2.71 times higher in the elderly compared to the young participants. Women have 2.234 times higher odds of visual impairment compared to men, while the odds of visual impairment were 14.83 times higher in the participants with the URE compared to the emmetropic participants. Almost two-thirds of participants (65.8%) reported never having had an eye examination before. Conclusions: Visual impairment was common in the community, especially among older people and females, and URE was the main cause of visual impairment, followed by amblyopia and cataract. Considerable subjects reported never having had an eye examination before; this highlights the challenges in accessing healthcare in rural areas. These findings emphasize the importance of improving healthcare accessibility in rural regions.

## 1. Introduction

Visual impairment can significantly impact an individual’s performance, productivity, and overall quality of life [1]. A recent study [2] reported that approximately 596 million people globally had VI, with 43.3 million of them being blind, and the majority of those affected resided in poor nations. The majority of causes leading to visual impairment can be prevented or treated through affordable interventions. According to the latest data from 2020, visual impairment resulted in an annual global productivity loss of approximately USD 410.7 billion [3,4]. According to recent estimates, the primary causes of blindness worldwide are cataracts, which affect 15.2 million individuals, followed by glaucoma (3.6 million), uncorrected refractive errors (URE) (2.3 million), age-related macular degeneration (1.8 million), and diabetic retinopathy (0.86 million). However, the main causes of mild visual impairment are UREs (86.1 million) and cataracts (78.8 million) [1,5,6].

UREs are a common cause of visual impairment; it is important to address UREs promptly, as they represent substantial causes of subtype visual impairment [1]. In addition, UREs became a priority condition targeted by the global initiative for the elimination of avoidable blindness, the Right to Sight Initiative. A high prevalence of URE or visual impairment in a community can serve as an indicator of the accessibility of healthcare services. A high prevalence of visual impairment often suggests inadequate access to healthcare resources within the community [1,7]. The World Health Organization (WHO) is launching a new initiative, SPECS 2030, to help countries achieve the global eye care target of a 40% increase in the proportion of individuals with access to appropriate spectacles. This initiative aims to reduce the causes of avoidable visual impairment, particularly uncorrected refractive errors [8].

In Saudi Arabia, several studies have reported the prevalence of visual impairment and URE. The prevalence of URE has varied from approximately 4.5% up to 45.8% [9,10,11,12,13,14,15,16], while the prevalence of visual impairment has been reported to be up to 24% [16]. The prevalence of visual impairment and URE in Saudi Arabia is considered relatively high [17]. However, most studies conducted in the country have primarily focused on urban communities or school children [8,9,10,11,12,13]. There is a lack of data on the prevalence of visual impairment and URE in rural areas, with only one recent study reporting the prevalence of visual impairment in a rural community [18]. This gap in research highlights the necessity for more studies to be conducted in rural areas to better understand the extent of visual impairment and associated risk factors in these communities. Therefore, this study aimed to report the prevalence, causes, and risk factors associated with visual impairment in Qbah, a rural community in the Qassim Province of Saudi Arabia.

## 2. Materials and Methods

### 2.1. Study Design and Setting

This descriptive cross-sectional study was conducted in Qbah, a rural community in the Qassim Province of Saudi Arabia. A sample of 587 subjects aged 6 to 80 were examined during the community eye health campaign.

### 2.2. Sample Size

The study was carried out during a community eye health campaign in a rural area of the Qassim region. The study sample included all 587 individuals who participated in the campaign. The campaign was a component of a larger medical mission to the community, offering eye care and other medical services. This annual event, organized by Qassim University, aims to provide healthcare to rural communities in Saudi Arabia.

### 2.3. Ethical Approval

The study received ethical approval from the Qassim University Health Research Ethics Committee (Number 19-7-06) and adhered to the guidelines outlined in the Declaration of Helsinki. Confidentiality of the collected data was maintained, ensuring no individual’s information was obtained. All individuals attending eye examinations were included in the study, with each subject or their guardian provided with a consent form for approval.

### 2.4. Clinical Investigation

Comprehensive eye examinations were performed, including visual acuity (VA), ocular health assessment, and non-cycloplegic refraction and subjective refraction. Qualified eye care professionals, including ophthalmologists and optometrists from Qassim University, conducted these examinations. Presenting visual impairment was classified based on the International Classification of Diseases 11th revision, 2018 (ICD-11). Mild VI is defined as visual acuity (VA) worse than 6/12 to 6/18, while moderate VI is VA worse than 6/18 to 6/60. However, severe VI is defined as VA worse than 6/60 to 3/60, and blindness is defined as VA worse than 3/60 [17]. Data collected included age, date of last eye examination, main complaint, VA, and refractive error (RE). In this study, RE was assessed objectively using a non-cycloplegic autorefractometer (NIDEK autorefractor (RK-310). RE was defined as follows: myopia as having at least -0.5 diopters (D) in one or both eyes, hyperopia as having at least +1.00 D in one or both eyes, and astigmatism as having 0.75 D or more cylindrical refraction. Additionally, slit lamp biomicroscopy was utilized to conduct an anterior eye examination. For the posterior segment examination of the eye, a direct ophthalmoscope and 90 D fundus biomicroscopy were used.

### 2.5. Data Analysis

The data were collected using Microsoft Excel, the information was edited and cleaned for missing values, and then all analyses were performed using SPSS statistical software (Version 24). Frequencies and percentages were employed to express categorical variables. The presence of RE, visual impairment, and its subtypes were assessed and analyzed descriptively using cross-tabulation. The prevalence of visual impairment and RE among participants by age group and gender was analyzed by MedCalc statistical software, version 23.1.7 or Windows. Additionally, risk factors for visual impairment, such as age, gender, date of last examination, and uncorrected refractive errors, were assessed using logistic regression and adjusted odds ratios (ORs) with a 95% CI. A significance level of *p*-value < 0.05 was used to determine statistical significance in all analyses.

## 3. Results

### 3.1. Vision Impairment and Demographic Features of the Participants

A total of 587 subjects were studied in the community study; their ages ranged from 6 to 80 years, with a mean age of 26.35 (18.48) years. The study included 372 (63.4%) males and 215 (36.6%) females. Most of the subjects, 386 (65.8%), reported never having had an eye examination before, while only 75 (12.8%) of the participants reported using eyeglasses. The visual impairment was highly associated with increased age and being female (*p* = 0.0001), as shown in Table 1.

### 3.2. Main Complaints Among Participants

Forty-five participants reported that they came for a check-up. Meanwhile, 29.3% of subjects complained of blurry vision, 6.3% reported headaches, 6.3% itching, 5.28% redness, 4.77% dry eyes, 2.73% tearing, and only 0.2% had squint.

### 3.3. The Prevalence of Visual Impairment Among Participants

In the present study, the overall prevalence of presenting visual impairment was 27.1% (95% CI: 23.4–31.6%). This included mild visual impairment (14.37%; 95% CI: 11.4–17.7), moderate visual impairment (11.7%; 95% CI: 8.6–14.1), severe visual impairment (0.90%; 95% CI: 0.30–0.21), and blindness (0.90%; 95% CI: 0.30–0.21), as shown in Table 2.

### 3.4. Prevalence of RE Among Participants

Table 3 showed the prevalence of RE in one or both eyes, categorized by age and gender. The overall prevalence of RE among participants was 321 (54.7% [95% CI, 48.9–61.1]). There was a statistically significant association between the prevalence of RE and both age and gender (*p* = 0.000) and (*p* = 0.002), respectively. Hyperopia, myopia, and astigmatism were prevalent in 134 (22.8% [95% CI, 19.1–27.4]), 174 (29.6% [95% CI, 25.4–34.4]), and 13 (2.2% [95% CI, 1.2–3.8]) subjects, respectively. The prevalence of myopia was more common in 18–39-year-old subjects (58; 43.3% [95% CI, 33.1–56.4]) and females (79; 36.7% [95% CI, 29.1–45.8]). Meanwhile, hyperopia was more prevalent in the age group of 60 and above (24; 55.8% [95% CI, 35.8–83.1]) and females (56; 26.1% [95% CI, 19.7–33.8]).

### 3.5. Risk Factors for the Visual Impairment

The regression analysis revealed that as age increased, the odds of visual impairment also increased. The odds of visual impairment were 2.71 times higher (95% CI: 1.84–3.98) in the 18–80 age group compared to the young participants aged 6–17 years. Women had 2.234 times higher odds (95% CI: 1.53–3.23) for visual impairment compared to men. Participants who underwent eye examinations for one year or more had 2.38 times higher odds (95% CI: 1.64–3.50) for visual impairment compared to those who never had their eyes checked. The odds for visual impairment were 14.83 times higher (95% CI: 8.30–26.52) in the participants with the URE group compared to the emmetropic participants, as shown in Table 4.

### 3.6. The Leading of Presenting Visual Impairment

In general, UREs (83.0%) were the main cause of visual impairment, followed by amblyopia (8.8%) and cataract (5.7%). UREs were the main cause of mild and moderate visual impairment, whereas cataract was the leading cause of severe visual impairment and blindness, as shown in Table 5.

## 4. Discussion

This study, conducted in Qbah, a rural community in the Qassim Province of Saudi Arabia, aimed to identify the prevalence, causes, and risk factors associated with visual impairment. The findings revealed that the overall prevalence of visual impairment was 27.1%; this included mild visual impairment (14.37%), moderate visual impairment (11.7%), severe visual impairment, and blindness (0.90%). URE was the main cause of visual impairment, at 83.0%, followed by amblyopia, at 8.8%, and cataract, at 5.7%. The overall prevalence of RE was 54.7%; this included hyperopia, myopia, and astigmatism, which were 22.8%, 29.6%, and 2.2%, respectively. Almost one-third of participants (29.3%) reported having blurry vision, and 6.3% reported experiencing headaches; however, 65.8% of the subjects reported never having had an eye examination before. Regression analysis indicated that as age increased, the odds of visual impairment also increased, emphasizing the importance of regular eye check-ups, especially among the elderly population. These significant findings underscore the need to improve eye care accessibility in rural areas like Qbah, as a majority of visual impairment cases are preventable with proper access to healthcare services.

The prevalence of visual impairment in the current study was found to be 27.1%. This figure aligns with another study conducted in rural areas that reported a visual impairment prevalence of 32.1% [16]. The slight difference in prevalence between the two studies could be attributed to differences in sample sizes. Conversely, a study conducted in Aljouf City, where individuals had better access to eye care, reported a visual impairment prevalence of 13.9% [19]. Despite focusing solely on adults, their sample size was similar to that of the current study. Moreover, the current findings revealed that the leading cause of visual impairment was URE, at 83.0%, followed by amblyopia, at 8.8%, and cataract, at 5.7%. Interestingly, another study conducted in urban cities of Qassim Province reported a significantly lower prevalence of amblyopia, at 3.90% [20]. This variance could be attributed to various factors, including access to primary eye care services. The comparison between studies conducted in urban and rural areas further supports this idea, as individuals in urban settings typically have better access to eye care services, consequently leading to a lower prevalence of visual impairment [16,19,20,21].

In the current findings, 54.7% of individuals were observed to have refractive errors (REs), including hyperopia (22.8%), myopia (29.6%), and astigmatism (2.2%). REs were defined as follows: myopia was classified as having at least -0.5 diopters (D) in one or both eyes, hyperopia as having at least +1.00 D in one or both eyes, and astigmatism as having 0.75 D or more cylindrical refraction. The prevalence of RE in Saudi Arabia showed a wide range, varying from 4.5% to 69.7% [8,9,10,11,12,13,14,15,16,17]. Some studies [9,15] indicated myopia as the most prevalent type, such as the prevalence of 29.6% observed in the current study. One study reported a prevalence of myopia of 14.3% in a pediatric age group [8], while the recent systematic review and meta-analysis of the prevalence of myopia among children in the Eastern Mediterranean Region reported a low prevalence rate of 5.23% [15]. On the other hand, some studies suggested that astigmatism was the most prevalent type [13,16]. These differences in the prevalence of RE could be due to geographical variations, as suggested by a previous study [13]. Furthermore, the differences could be due to the benchmark used for the definition of RE or the methods used for measuring RE: some studies assessed the refraction under cycloplegia, while others assessed it without cycloplegia. The present study reported a high prevalence of myopia, which could be attributed to environmental factors, such as a lack of outdoor activities, as many young individuals are engaged in near tasks. Additionally, genetic predispositions may play a role, as consanguineous marriages are common in the Saudi community. 

Amblyopia is a preventable and reversible cause of visual impairment and is particularly manageable with early and proper eye care. In the current study, amblyopia was responsible for VI in 8.8%, whereas the global prevalence of amblyopia among children is relatively low (1.36%) [22]. A study conducted in the Qassim region of Saudi Arabia among children showed that the commonest types of amblyopia in children were attributed to strabismus and anisometropia and commonly found in males [23]. This high occurrence of amblyopia suggests a lack of access to primary eye care services in this rural community. It is concerning that 65.8% of the subjects reported never having undergone an eye examination before, and the nearest eye care facility being 150 km away from Qbah stresses the challenges of accessing healthcare in rural areas. These study findings emphasize the critical need to address healthcare accessibility gaps in rural regions. Given the ongoing healthcare reforms in Saudi Arabia and the goals set for vision 2030, such studies and findings highlight crucial healthcare issues and can help drive initiatives aimed at positive changes in healthcare accessibility and quality. Diabetic retinopathy contributes to 2.5% of visual impairment within this ruler community. Consequently, the current study proposes that future research should focus on evaluating the impact of diabetes and hypertension on ocular health among the population in Saudi Arabia.

To address the high prevalence of visual impairment in rural areas, improving access to eye care is essential. Mobile eye clinics, community outreach, and school-based screenings can help detect and treat visual impairments early. Public health campaigns should raise awareness, particularly for high-risk groups like older adults, women, and individuals with systemic conditions such as diabetes and hypertension. Policymakers should prioritize subsidized eye care and foster public–private partnerships to improve accessibility and affordability. Further research is needed to investigate barriers to accessing eye care in rural areas and explore the influence of genetic and environmental factors on myopia development. Furthermore, the study proposes forming partnerships with local healthcare providers and NGOs in Saudi Arabia to bridge gaps in healthcare accessibility and implement sustainable interventions. Longitudinal studies can provide insights into the effect of visual impairment and its long-term effects on quality of life. Additionally, evaluating the effectiveness and cost-efficiency of interventions, such as mobile clinics and health education programs, will guide resource allocation and policy development. These steps will support the development of targeted strategies to reduce visual impairment and improve eye health outcomes in underserved communities.

Despite the importance of these findings, there are some limitations that need to be addressed. Firstly, the inability to perform cyclorefraction in the pediatric age group may lead to under- or over-detection for certain types of refractive errors [24]. Secondly, the benchmark for definition of hyperopia in the pediatric population is typically considered to be +2.00 D, yet in the current study, it was set at +1.00 D due to the diverse range of ages included in the sample. This could potentially result in an overestimation of hyperopia in children under the age of 7. Furthermore, people who come to the camp may have a complaint; this could increase the prevalence rate reported in the present study. In the present study, we addressed uncorrected refractive errors in detail and less detail in other causes; this could be due to the use of an autorefractometer, which gives a very accurate objective measurement of refractive error. Despite the limitations mentioned above, the present study highlighted important information regarding the prevalence, causes, and risk factors associated with visual impairment in the rural community of Saudi Arabia.

## 5. Conclusions

Visual impairment was common in the community, especially among older people and females, and URE was the main cause of visual impairment, followed by amblyopia and cataract. A significant proportion of subjects (65.8%) reported never having undergone an eye examination before. These findings highlight healthcare-access challenges and advocate for initiatives aimed at improving healthcare quality and accessibility. Moreover, addressing issues such as URE and improving access to primary eye care services such as mobile eye care units or subsidized corrective eyewear programs could effectively tackle the identified challenges and potentially alleviate the burden of visual impairment in rural communities.

## Figures and Tables

**Table 1:** Visual impairment and demographic features of the participants.

Characteristics			*p*-Value
Age Group (Years)	Total n (%)	Visual Impairment, n (%)	<0.001
6–10	122 (20.8)	15 (12.3)	
11–17	165 (28.1)	35 (21.2)	
18–39	133 (22.7)	34 (25.6)	
40–49	78 (13.3)	25 (32.1)	
50–59	46 (7.8)	20 (43.5)	
60 and above	43 (7.3)	30 (69.8)	
Gender			<0.001
Male	372 (63.4)	79 (21.2)	
Females	215 (36.6)	80 (37.2)	
Date of last examination			<0.001
One year or more	201 (34.2)	78 (38.8)	
Never	386 (65.8)	81 (21.0)	
Wearing glasses			<0.001
Yes	75 (12.8)	49 (65.3)	
No	512 (87.2)	110 (21.5)	
Total	587	159 (27.1) [95%CI, 23.4–31.6]	

**Table 2:** The prevalence of visual impairment among participants by age groups and gender.

Characteristics	Presenting Visual Impairment					Total
	Mild Visual Impairment n (%)	Moderate Visual Impairment n (%)	Severe Visual Impairment n (%)	Blindness n (%)	NAD n (%)	
Age (years)	*p* < 0.001					
6–10	13 (10.7)	2 (1.64)	0 (0.0)	0 (0.0)	107 (87.7)	122
11–17	18 (10.9)	15 (9.1)	1 (0.6)	1 (0.6)	130 (78.8)	165
18–39	13 (9.8)	18 (13.5)	2 (1.5)	1 (1.0)	99 (74.4)	133
40–49	13 (16.7)	10 (12.8)	1 (1.3)	1 (1.3)	53 (67.9)	78
50–59	12 (26.1)	8 (17.4)	0 (0.00)	0 (0.0)	26 (56.5)	46
60 and above	15 (34.9)	12 (27.9)	1 (2.3)	2 (4.7)	13 (30.2)	43
Gender	*p* < 0.001					
Male	47 (12.6)	26 (7.0)	3 (0.8)	3 (0.8)	2 (49.9)	372
Female	37 (17.2)	39 (18.1)	2 (0.9)	2 (0.9)	135 (62.8)	215
Total	84 (14.3)	65 (11.7)	5 (0.9)	5 (0.9)	428 (72)	587
95% CI	14.3, 11.4–17.7	11.7, 8.6–14.1	0.9, 0.30–0.21	0.9, 0.30–0.21	72, 66.2–80.2	

**Table 3:** The prevalence of RE among participants by age groups and gender.

Characteristics	Refractive Error 321 (54.7% [95% CI, 48.9–61.1])				Total
	Emmetropia n (%)	Hyperopia n (%)	Myopia n (%)	Astigmatism n (%)	
Age (years)	*p* < 0.001				
6–10	67 (54.9)	21 (9.8)	30 (24.6)	4 (3.3)	122
11–17	88 (53.3)	23 (13.9)	51 (30.9)	3 (1.8)	165
18–39	58 (43.6)	13 (9.8)	58 (43.6)	4 (3.0)	133
40–49	34 (43.6)	30 (38.5)	14 (17.9)	0 (0.0)	78
50–59	13 (28.3)	23 (50.0)	9 (19.6)	1 (2.2)	46
60 and above	6 (14.0)	24 (55.8)	12 (27.9)	1 (2.3)	43
Gender	*p* = 0.002				
Male	190 (51.1)	78 (12.3)	95 (25.5)	9 (2.4)	372
Female	76 (35.3)	56 (26.1)	79 (36.7)	4 (1.9)	215
Total	266 (45.3)	134 (22.8)	174 (29.6)	13 (2.2)	587
95% CI	45.3, 40.0–51.1	22.8, 19.1–27.4	29.6, 25.4–34.4	2.2, 1.2–3.8	

**Table 4:** Effects of demographic features and RE in visual impairment among participants (multinomial logistic regression analysis).

Characteristics	Adjusted Odds Ratio (95% CI)	*p*-Value
Age group (years)		
6–17	Reference	
18–80	2.71 (1.84–3.98)	<0.001
Gender		
Male	Reference	
Female	2.23 (1.53–3.23)	<0.001
Date of last examination		
Never	Reference	
One year or more	2.38 (1.64–3.50)	<0.001
Refractive error (RE)		
Emmetropia	Reference	
Uncorrected RE	14.83 (8.30–26.52)	<0.001

**Table 5:** The main causes of presenting visual impairment.

Causes	Presenting Visual Impairment				Total n (%)
	Mild Visual Impairment n (%)	Moderate Visual Impairment n (%)	Severe Visual Impairment n (%)	Blindness n (%)	
Refractive error	77 (91.7)	55 (84.6)	0 (0.0)	0 (0.0)	132 (83.0)
Amblyopia	6 (7.1)	6 (9.2)	1 (20.0)	1 (20.0)	14 (8.8)
Cataract	0 (0.0)	3 (4.6)	3 (60.0)	3 (60.0)	9 (5.7)
Diabetic retinopathy	1 (1.2)	1 (1.5)	1 (20.0)	1 (20)	4 (2.5)
Total	84 (100)	65 (100)	5 (100)	5 (100)	159 (100)

## Data Availability

There are no additional data deposited on any other site other than in this manuscript.

## Abbreviations

The following abbreviations are used in this manuscript:

ICD-11	International Classification of Diseases 11th revision
UREs	uncorrected refractive errors
VA	visual acuity
RE	refractive error
